# Exploring public preferences and demand for ovarian cancer screening: a discrete choice experiment

**DOI:** 10.3389/fonc.2025.1467457

**Published:** 2025-04-24

**Authors:** Rebekah Hall, Anne E. Spencer, Abigail Lloyd, Willie Hamilton, Antonieta Medina-Lara

**Affiliations:** University of Exeter Medical School, University of Exeter, Exeter, United Kingdom

**Keywords:** ovarian cancer_1_, screening_2_, discrete choice experiment_3_, preferences_4_, demand_5_

## Abstract

**Introduction:**

Routine population-level screening may in the future reduce the high mortality rates associated with late-stage ovarian cancer diagnosis. However, the voluntary nature of screening means that understanding the public’s acceptability of the benefits, harms and likely uptake of any potential screening programme is crucial to implementation.

**Objective:**

To measure public preferences towards the benefits and harms of a potential screening programme and to predict uptake.

**Methods:**

An online Discrete Choice Experiment was completed by 250 women 40-80 years old in England and Wales. Subjects were asked 12 questions where they were asked to choose between two hypothetical screening tests described in terms of four attributes; ovarian cancer deaths, false-positive, false-negative and overdiagnosis rates, and no screening. Responses were analysed using mixed logit regression.

**Results:**

In total, 250 women completed the survey. Ovarian cancer deaths (0.42, [95% CI: 0.40 – 0.44]) was the most important attribute overall, followed by the rate of false positive results (0.30, [95% CI: 0.30-0.30]). However, there were high levels of heterogeneity with individuals exhibiting low levels of worry about ovarian cancer (OR=1.76 [95% CI: 1.17–2.69]), low perceived risk of ovarian cancer (OR=1.44 [95% 1.03–2.03]) or risk-averse individuals (OR=1.46 [95% CI: 1.05–2.04]) significantly more likely to opt for the no screening alternative. Oppositely, individuals who regularly participate in cervical screening (OR=0.63 [0.47–0.90]) were less likely to opt for no screening. Overall, results indicated participants would be willing to accept 2.59 (95% CI: 1.82 – 3.36) false-negative results, 205 (95% CI: 161 – 248) false-positive results and 2.35 (95% CI: 1.76-2.94) per 10,000 people screened to avoid 1 ovarian cancer-related death. Uptake analysis confirmed a high willingness to undergo screening across varying levels of benefits and harms.

**Conclusions:**

Currently ovarian cancer screening is not recommended as available screening methods do not offer benefits in terms of mortality reduction. The results of this study demonstrate a high demand for ovarian cancer screening and a willingness to trade between the benefits and risks of a potential test. Results of this study provide a useful resource for assessing the acceptability of future screening modalities which may become available.

## 1 Introduction

Ovarian cancer is the 8^th^ most common cancer in females worldwide, with over 300,000 new cases diagnosed annually, accounting for 4% of all new cancer cases in females (1). Symptoms of ovarian cancer are non-specific and present in many other conditions; furthermore, public awareness of these symptoms remains low, making early diagnosis a challenge. However early diagnosis is important to survival; five-year survival for women diagnosed with stage IV cancer is just 13%, as opposed to 93% for women diagnosed at stage I (2).

Effective cancer screening programmes increase survival through detection of pre-cancerous or early-stage disease in asymptomatic individuals (3). Given the challenges to early diagnosis for ovarian cancer, screening in populations before symptoms arise may be a solution to reduce late-stage diagnoses and ultimately save lives. There have been efforts to identify an appropriate ovarian cancer screening programme for over three decades, with multiple national and international clinical trials taking place in both average-risk and high-risk populations (4). To date, trials have demonstrated existing screening methods offer no benefits in terms of survival and are associated with several harms, in particular high levels of false positive results with unnecessary follow up testing (3). However, given the high mortality rates associated with ovarian cancer, research to develop an appropriate screening programme is ongoing (3, 4).

Fundamentally, any screening programme must demonstrate an ability to reduce deaths or disease incidence whilst remaining cost-effective. Given the voluntary nature of screening participation, governing bodies such as the National Screening Committee (NSC) in the UK also stipulate that any screening programme is clinically, socially and ethically acceptable to the public and that the benefit gained by individuals from the screening programme should outweigh any harms (5). As more concerted efforts are made to provide frameworks for minimum optimal performance characteristics for new diagnostic tests (6) and product development in health technology assessments (7), it is also likely that patients preferences and uptake will be central to developments of these for screening and diagnosis.

To date, research on preferences for ovarian cancer screening has typically focused on the perspective of patients enrolled on screening trials (8–10). However, evidence suggests these individuals are more likely to have a favourable view of screening and exhibit a willingness to be screened regardless about beliefs surrounding the efficacy of screening, meaning results may not be representative of the wider public (11, 12). In contrast, there is limited information available on the preferences of general public, average-risk individuals, despite them being the target population for a potential screening programme.

Moreover, studies of acceptability have typically focused on factors relating to test experience (e.g. test modality, pain, healthcare provider characteristics) (13, 14). In comparison, preferences and acceptability of test performance characteristics such as the numbers of false-positives, false-negatives and overdiagnosis remain underexplored. NSC recommendations are in part determined by the balance between benefits and harms of screening; however, the acceptable balance is currently unknown for ovarian cancer.

In response to these unexplored issues, the purpose of this study was to investigate women’s preferences and demand for a potential screening programme for the target population using an online survey with an embedded Discrete Choice Experiment (DCE). The study explored the acceptable balance of benefits and harms of hypothetical tests which could be potentially used to screen for ovarian cancer before symptoms arise. Ethical approval for this study was granted by the University of Exeter Medical School Research Ethics Committee (Research Ethics Committee approval reference number: Oct20/B/26).

## 2 Methods

An online survey with a DCE component was designed to understand the screening preferences of women living in England and Wales over 40 years old. A full copy of the survey is provided in **Supplementary Material 1**. The survey was split into seven sections. In the first section participants were provided with introductory information relating to ovarian cancer and screening. The next section included questions to measure participants’ knowledge and experience of ovarian cancer (e.g. symptom awareness). Section 3 contained the DCE component of the survey. This included an introduction to the attributes and a practice question. Section 4 consisted of debriefing questions about the DCE task including task difficulty. Next, section 5 contained standardized sociodemographic questions such as employment status, education level and age. Section 6 included questions relating to health beliefs and behaviour (e.g. current screening behaviour). The final section consisted of numerical ability questions. Where possible questions were taken from validated sources such as national surveys (e.g. UK census).

### 2.1 Discrete choice experiment

DCEs quantify strength of preference by presenting participants with a series of choices tasks where they must state their preference between two or more hypothetical screening tests. Each test was described in terms of key characteristics (“attributes”), each of which may consist of a number of variations (“levels”). Analysing the choices of participants allows the relative importance and trade-offs between different test characteristics to be estimated in situations that cannot be routinely observed (15).

### 2.2 Attributes and levels

Attribute selection utilised an iterative multi-method approach. An initial longlist of thirteen attributes was identified through a systematic review of published DCEs eliciting preferences towards cancer screening (16) and a targeted review of qualitative literature relating to cancer screening. Next, attributes were reduced based on a Best-Worst Scaling study with 100 members of the target population (17).

The BWS results demonstrated a clear prioritisation of the four test performance attributes included in this study: false positive results, false negative results, overdiagnosis and number of ovarian cancer deaths. Additional attributes were omitted to manage participant burden and because cognitive debriefing suggested inclusion of additional service delivery attributes (e.g. test modality, duration, location etc.) could lead to substantial attribute non-attendance. This focus on test performance characteristics allowed the balance of risks and benefits associated with cancer screening to be fully explored.

Attributes were tested and refined through five cognitive debriefing interviews with five women aged 43-65 years old living across England. Interviews lasted between 35 and 52 minutes.

A review of clinical trial evidence formed the basis of attribute levels; however, limited success of ovarian cancer screening to date meant level ranges were extended beyond currently observed efficacy levels to allow benefits of potential future screening tests to be evaluated (4). Finalized attributes and levels are shown in **Table 1**. At the start of the questionnaire the attributes were described in more detail. In particular, information was given on the requirement for unnecessary surgery when receiving a false positive result in ovarian cancer screening, the potential for false reassurance following a false negative result and the potential for unnecessary treatment in overdiagnosis (see **Supplementary Material 1**). For example, false positives were described as follows: *“These are people who do not have cancer but receive a positive (or abnormal) result. People who receive an incorrect positive result will undergo unnecessary, often invasive testing. A small proportion of these people (about 3%) will undergo unnecessary surgery because of the incorrect result.”*

**Table 1:** Description of attributes and levels.

Attribute wording	Definition	Levels
Ovarian cancer deaths	The number of people who will die of ovarian cancer over the course of 10 years	10 per 10,000 people screened 20 per 10,000 people screened 30 per 10,000 people screened 40 per 10,000 people screened (No screening)
False positive results	The number of people who do not have cancer that will receive an incorrect positive result over the course of 10 years	0 per 10,000 people screened (No screening) 1,000 per 10,000 people screened 2,000 per 10,000 people screened 3,000 per 10,000 people screened 4,000 per 10,000 people screened
False negative results	The number of people with cancer who will receive an incorrect negative result over the course of 10 years	0 per 10,000 people screened (No screening) 3 per 10,000 people screened 7 per 10,000 people screened 10 per 10,000 people screened 13 per 10,000 people screened 16 per 10,000 people screened 20 per 10,000 people screened
Overdiagnosed cancers	The number of people who will be unnecessarily diagnosed and treated for cancer that would never have killed them or even caused symptoms over the course of 10 years	0 per 10,000 people screened (No screening) 3 per 10,000 people screened 7 per 10,000 people screened 10 per 10,000 people screened 13 per 10,000 people screened 16 per 10,000 people screened

### 2.3 Experimental design and choice tasks

A full-factorial design would result in 3^1^ x 4^1^x 6^2^ = 432 choice tasks and 93,096 potential paired combinations. Instead, a Bayesian efficient fractional factorial design was generated using Ngene 1.2 (Choice Metrics) using results from a quantitative pilot study (n=40) to inform priors. The final design included 12 tasks. An additional task that contained a dominant alternative was included as a warmup task meaning participants completed 13 choice tasks in total.

**Figure 1:**
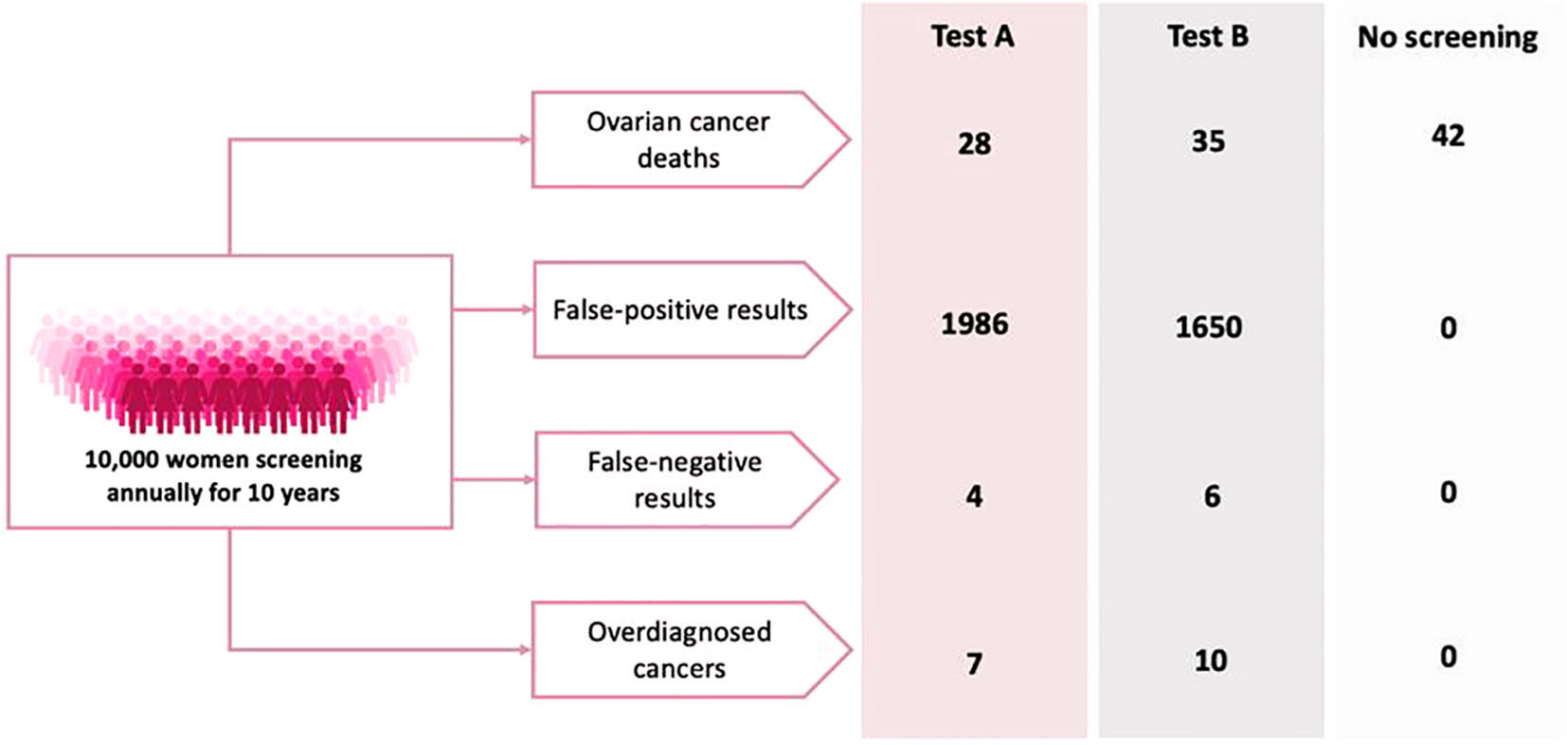
An example DCE task.

An example task is shown in **Figure 1**. Each choice task included two unlabelled test options and a “no screening” alternative. Communication of small risks was a primary challenge during the development of the DCE. Alternative risk presentation options were designed based on published guidance and adaptations of existing decision aids (17). The alternative versions were tested in an online questionnaire with 50 women over the age of 40. Participants were shown all four versions and asked which they preferred—specifically, which they found easiest to understand. An adapted probability tree was selected for this study as it was most favoured by the target population and subsequent cognitive debriefing interviews suggested the format was well understood. Given the low prevalence of ovarian cancer, all probabilities were converted from 100,000 patient years to per 10,000 patients in a 10-year period to aid participant understanding.

### 2.4 Study population and recruitment

The target population for the DCE was women with at least one ovary over the age of 40 years old living in the UK. A general public population female sample was selected since this would be the target population eligible for screening if/when universal screening becomes available. A minimum sample size of 45 participants was estimated based on the s-estimate approach proposed by (18). A final sample size of 250 was selected to allow exploration of preference heterogeneity.

Participants were recruited using Prolific (Prolific.co), an online recruitment platform. Participants were screened based on sex and age initially, with a follow up question designed to identify people without ovaries who were ineligible for inclusion. Any account holder who met the eligibility criteria was notified of the study by Prolific. The eligible population was identified using the filter options available on Prolific. Recruitment into the study was performed on a first-come, first-served basis until the target number of responses was met. Participants who were interested in taking part were then given further information about the study and completed an online consent form prior to beginning of any study questions. Individuals who decided not to proceed were redirected to an exit page and thanked for their time. Participants who completed the survey were compensated at a rate of £8 per hour. Given the length of the survey and online administration method, three attention checks following the instructional manipulation format were embedded (e.g. ‘Select ‘very important’ to indicate you are paying attention’) (19). Participants who failed all three attention checks were removed from the analysis.

### 2.5 Analysis

Demographic characteristics were descriptively summarized using means and standard deviations. Preference data was analysed using Stata 17.0. A main-effects multinomial logit (MNL) model including dummy-coded attribute levels was estimated. Parameter coefficients were assessed to determine the functional form of each attribute. Following confirmation of the correct functional form, a mixed logit (ML) model was estimated to account for unobserved preference heterogeneity among participants. Ultimately, a main-effects model with a continuous linear specification for all attributes was estimated based on the following utility function:


$$\begin{array}{c} V=\alpha_{B}+\;\alpha_{Opt-out}+\beta_{1}\text{mortality}\\ +\beta_{2}\text{falsenegative}\;+\beta_{3}\text{falsepositive}+\beta_{4}\text{overdiagnosis} \end{array}$$

Beta coefficients, $\beta_{1}-\beta_{4}$ represent the relative utility weights of the four test performance attributes. The opt-out alternative was incorporated in the model using an alternative specific constant (ASC), $\alpha_{Opt-out}$ . An additional ASC, $\alpha_{B}$ was included to account for any left-right bias in participant choices.

Results from the ML model were used to calculate relative attribute importance (RAI) by dividing each attribute’s marginal utility range by the sum of the utility ranges across all attributes. RAI represents the proportion of a screening alternative’s utility that can be attributed to changes in each attribute. The Delta method was applied to obtain the 95% confidence interval around estimates (20).

Utility weights were also used to calculate the willingness to trade between the potential benefits and harms of screening. Specifically, the willingness to accept (WTA) additional risks (i.e. overdiagnosed cancers, false positives or false negatives) to avoid one extra death. Equation 1 provides an example WTA calculation. Results are interpreted as the additional number of false negative results per 10,000 people screened that would be accepted in exchange for one ovarian cancer death avoided over a 10-year period.


(1) $$\frac{\beta_{1}\text{mortality}}{\beta_{2}\text{falsenegative}}$$

Finally, utility weights were used to explore the expected demand for screening. As no screening programme for ovarian cancer is currently available, scenarios were designed to assess variations in demand based on varying combinations of test characteristics for demonstrative purposes. For each scenario, the probability of accepting the screening alternative compared to no screening was calculated using the following formula:


$$P_{screening}=\;\frac{e^{\left[V_{screening}\right]}}{\left(e^{\left[V_{screening}\right]}+\;e^{\left[V_{no\;screening}\right]}\right)}$$

Where $V_{screening}$ is the expected utility for the screening scenario and $V_{\;no\;screening}$ is the expected utility for the no screening alternative.

#### 2.5.1 Analysis of opt-out behaviour

Selection of the “no screening” alternative was examined to identify any common characteristics associated with increased non-screening behaviour. Reasons for serial non-participation across all choice tasks were summarized narratively. Finally, a logistic regression model with opt-out choices as the dependent variable and sociodemographic characteristics as the independent variables was estimated.

## 3 Results

Responses were collected in January 2022. In total, 258 individuals began the survey. Four people dropped out part way through and an additional 4 people were excluded after failing the attention check questions, leaving a final sample size of 250 participants.

### 3.1 Sociodemographic and behavioural characteristics

Key participant characteristics are summarized in **Table 2**. Participants ranged from 40 to 80 years old with an average age of 53 years. Most participants were white (n=198, 79%) and university educated (n=138, 55%). On average, participants leaned towards risk aversion (mean = 4.3, [SD=2.2]). Responses to key health-related questions are provided in **Supplementary Material 3**. Ovarian cancer worry was generally low across the population, with 74% of participants stating little-to-no worry (184/250). Over three-quarters of participants (192/250; 77%) indicated that they were not confident in their ability to recognise symptoms of ovarian cancer. Rates of symptom recognition ranged from 22% (55/250) to 68% (169/250), and 12% (29%) did not recognise any key ovarian cancer symptoms when provided a list. Current screening behaviour was varied; however, 63% (157/250) of participants reported undergoing cervical screening every time they received an invitation.

**Table 2:** Sociodemographic characteristics of participants completing the DCE survey.

Characteristic	
Age	
Mean (SD)	52.9 (8.7)
Range	40-80
Ethnicity, n (%)	
White	198 (79%)
Mixed-white and black Caribbean	6 (2%)
Mixed- white and Asian	5 (2%)
Asian- Indian	5 (2%)
Asian- Chinese	8 (3%)
Black- African	9 (4%)
Black- Caribbean	10 (4%)
Other	6 (2%)
Prefer not to say	3 (1%)
Children	
Mean (SD)	1.52 (1.2)
Range	0-5
Relationship status, n (%)	
Single	36 (14%)
In a relationship	46 (18%)
Married/civil partnership	125 (50%)
Separated/divorce	34 (14%)
Widowed	9 (4%)
Education, n (%)	
No qualifications	2 (1%)
GCSE	53 (21%)
A-Level/ College	51 (20%)
Undergraduate	86 (34%)
Post-graduate/ professional quals	52 (21%)
Other	4 (2%)
Prefer not to say	1 (0.4%)
Employment, n (%)	
Employed, full-time	84 (34%)
Employed, part-time	58 (23%)
Self-employed	35 (14%)
Not employed	9 (4%)
Retired	31 (12%)
Other	31 (12%)
Prefer not to say	2 (1%)
Willingness to take risks (1 not at all – 10 completely willing)	
Mean (SD)	4.3 (2.2)
Task difficulty, n (%)	
Very easy	11 (4%)
Easy	59 (24%)
Neither easy or difficult	63 (25%)
Difficult	104 (42%)
Very difficult	13 (5%)

### 3.2 Preference results

A multinomial logit model using dummy-coded levels was initially estimated to check the functional form of all attributes. Coefficient plots for each attribute were examined and continuous linear coding appeared to be acceptable based on visual inspection.

In total, twelve participants chose the inferior alternative in the dominance rationality check choice task. Sensitivity analysis revealed no significant changes in any model parameters when failing participants were excluded from the analysis (**Supplementary Material 4**). On this basis, all responses were included in the analysis.

**Table 3** shows the results of the final mixed logit model. All attributes were significant and followed the expected direction. Since all attributes were negatively framed, the negative coefficients indicate an increase in incidence; for example, additional people dying from ovarian cancer leads to a reduction in utility associated with screening. The alternative specific constant associated with the “no screening” alternative was negative and large in magnitude, demonstrating an overall preference to be screened. However, the large standard deviation (4.74) indicates high levels of heterogeneity across participants with almost 40% of participants showing a preference towards no screening (based on a z-score of 2.29/4.74 = 0.48). Heterogeneity in preferences across the remaining parameters was also observed, but at much lower levels as indicated by the smaller standard deviations.

**Table 3:** Mixed logit results.

	Mean (95% CI)	SD	Relative importance	Willingness to accept per 1 additional death avoided (95% CI)
Ovarian cancer deaths	-0.14*** (-0.16 – [-0.12])	0.10*** (0.08 – 0.12)	0.42 (0.40 – 0.44)	–
False negative results	-0.05*** (-0.07 – [-0.04])	0.05*** (0.03 – 0.07)	0.14 (0.12 – 0.15)	2.59 (1.82 – 3.36)
False positive results	-6.81x10^-4^*** (-8.10x10^-4^ – [-5.52x10^-4^])	6.53x10^-4^*** (4.91x10^-4^ – 8.16x10^-4^ )	0.30 (0.30 – 0.30)	205.20 (161.89 – 248.51)
Overdiagnosed cancers	-0.06*** (-0.07 – [-0.05])	0.04*** (-0.18 – 0.35)	0.14 (0.13 – 0.15)	2.35 (1.76 –2.94)
No screening	-2.29*** (-3.01 – [-1.57])	4.74*** (3.75 – 5.72)	–	–
LL	-1913.08			
LR test (ML vs MNL)	1937.2***			
Observations	9,000			
N	250			

***significant at 99% confidence level.

Relative importance scores for each attribute are shown in **Table 3**. Ovarian cancer deaths (0.42, [95% CI: 0.40 – 0.44]) was most important overall, followed by the rate of false positive results (0.30, [95% CI: 0.30-0.30]).

#### 3.2.1 Willingness to accept additional risks in exchange for lives saved

**Table 3** shows the willingness to accept extra harms of testing to avoid one additional ovarian cancer death. Results relate to the number of additional harms per 10,000 people screened over a 10-year period. For example, participants were willing to accept an additional 205 false positive results over 10 years in exchange for one life saved.

#### 3.2.2 Predicted demand for screening

**Table 4** shows the estimated uptake rates for a series of hypothetical screening tests with different characteristics. Results further demonstrate a high intention to undergo screening even where potential harms from screening are high and reduction in mortality is moderate.

**Table 4:** Estimated uptake of hypothetical ovarian cancer screening test with varying performance characteristics.

	Performance characteristics*				Predicted uptake
	Ovarian cancer deaths	False negatives	False positives	Overdiagnosis	% Participation (95% CI)
1	36 in 10,000 (10% reduction)	14 in 10,000 (20%)	2981 in 10,000 (3%)	3 in 10,000 (5%)	47.1% (30.2 – 64.0%)
2	30 in 10,000 (25% reduction)	23 in 10,000 (35%)	994 in 10,000 (1%)	28 in 10,000 (30%)	52.6% (34.5 – 70.7%)
3	36 in 10,000 (10% reduction)	7 in 10,000 (10%)	1987 in 10,000 (2%)	0 in 10,000 (0%)	75.3% (63.1 – 87.5%)
4	30 in 10,000 (25% reduction)	3 in 10,000 (5%)	1987 in 10,000 (2%)	7 in 10,000 (10%)	85.2% (77.0 – 93.5%)
5	10 in 10,000 (75% reduction)	14 in 10,000 (10%)	3974 in 10,000 (4%)	17 in 10,000 (20%)	88.2% (80.0 – 96.7%)
6	20 in 10,000 (50% reduction)	7 in 10,000 (10%)	1987 in 10,000 (2%)	0 in 10,000 (0%)	96.6% (94.3 – 99.0%)
7	10 in 10,000 (75% reduction)	10 in 10,000 (15%)	2981 in 10,000 (3%)	3 in 10,000 (5%)	97.7% (95.8 – 99.5%)
8	20 in 10,000 (50% reduction)	7 in 10,000 (10%)	497 in 10,000 (0.5%)	0 in 10,000 (0%)	98.7% (97.8 – 99.7%)
9	8 in 10,000 (80% reduction)	3 in 10,000 (5%)	994 in 10,000 (1%)	0 in 10,000 (0%)	99.7% (99.4 – 99.9%)
10	0 in 10,000 (100% reduction)	0 in 10,000 (0%)	3974 in 10,000 (4%)	17 in 10,000 (25%)	99.1% (98.3 – 99.9%)

*Percentages shown in brackets are based on an incidence of 65 cases per 10,000 females and mortality rate of 40 per 10,000 females control arm of the UKCTOCS trial [Menon et al, (42)].

#### 3.2.3 Opt-out behaviour

The no screening alternative was selected in 10% of all choice tasks (929/9000); 109 (44%) participants opted not to be tested in at least one instance. Logistic regression found individuals who considered themselves low risk (OR=1.48) or exhibit low levels of worry about ovarian cancer (OR=1.76) were significantly more likely to select the no screening alternative (**Table 5**). Risk-averse individuals were also more likely to opt-out (OR 1.45) Oppositely, individuals who regularly participate in cervical screening were less likely to opt for no screening (OR=0.63).

**Table 5:** Logistic regression results exploring the relationship between sociodemographic characteristics and selection of the "no screening" alternative.

	Full model	Reduced model
	Odds ratio (95% CI)	Odds ratio (95% CI)
Age	1.01 (0.99–1.03)	
Employed	0.75 (0.53–1.07)	
Ethnicity- white	0.77 (0.48–1.24)	
Number of children	0.94 (0.81–1.10)	
Attended university	1.08 (0.77–1.51)	
Know someone diagnosed with ovarian cancer	0.77 (0.46–1.29)	
Always attends cervical screening	0.66*** (0.48–0.92)	0.65** (0.47–0.90)
Found DCE difficult/very difficult	0.90 (0.65–1.23)	
Low ovarian cancer worry	1.68** (1.11–2.53)	1.77** (1.17–2.69)
Low perceived ovarian cancer risk	1.33* (0.92–1.93)	1.44** (1.03–2.03)
Numerical ability	1.00 (0.83–1.19)	
Self-reported health: Very good—good	1.20 (0.81–1.76)	
Number of symptoms recognised	0.99 (0.94–1.04)	
Risk averse	1.52** (1.06–2.18)	1.46** (1.05–2.04)
Low confidence in ability to recognise OC symptoms	0.72* (0.51–1.03)	0.73** (0.52–1.02)
Constant	0.08 (0.02–0.30)	0.09*** (0.05–0.14)
Model fit statistics		
LL	-2885.68	-2908.33
Pseudo R^2^	0.03	0.03
N	250	250

***signifies a p-value<0.01; **significant at 95% confidence level; *significant at 90% confidence level.

## 4 Discussion

This study quantifies preferences relating to the benefits and harms of potential future screening tests for ovarian cancer, with a particular focus on test performance characteristics. The results provide a basis for understanding the minimum requirements for acceptability and expected uptake rates for potential future ovarian screening programmes.

Mixed logit results revealed the number of ovarian cancer deaths was considered the most important attribute overall, followed by the rate of false positive results. Overdiagnosed cancers and false negative results appeared to be similar but of lower importance. Willingness to accept analyses revealed participants would trade additional harms in exchange for the benefit of reductions in ovarian cancer mortality.

Although our results generally indicate a strong overall preference for screening, mixed logit estimates demonstrated significant heterogeneity in preferences. Those who were employed and those who regularly attended cervical screening were less likely to decline screening, whereas those who considered themselves at low risk of ovarian cancer, those experiencing low levels of worry about ovarian cancer, and risk averse individuals were more likely to opt for no screening. However, the overall explanatory power of the model was low, suggesting opt-out decisions may be more subtly motivated. Qualitative analysis of reasons for serial non-testers further confirmed a low perceived risk of ovarian cancer to be a key driver of choosing to forgo screening alongside attribute-driven reasons (e.g. unacceptable risk-benefit ratios).

### 4.1 Key implications

#### 4.1.1 Preferences towards risk benefit trade offs

The evidence from this study addresses key criteria considered by regulatory bodies such as the UK National Screening Committee when assessing potential national screening programmes, by providing a valuable reference for assessment if and when candidate tests emerge (46). Specifically, this study provides evidence on the acceptability of a potential screening programme and the balance between benefit and harms from a public perspective. Crucially, participants demonstrated a willingness to trade between the benefits and harms of ovarian cancer screening.

#### 4.1.2 Demand for screening

Uptake analysis demonstrated high intention to undergo screening where even a small reduction in mortality was expected and the risk of potential harms of testing were high. The reasons for opting out provides key insights into how screening uptake may be optimized, by revealed key characteristics that may drive screening decisions beyond test characteristics. On the other hand, the relationship between reduced participation in cervical cancer screening and intentions to be screened for ovarian cancer implies for some, there may be a more fundamental attitude against screening in general.

#### 4.1.3 Policy implications

Findings from this study have important implications for current practice. Surveys of GPs have found that *ad hoc* screening of low-risk women is not uncommon with approximately 30% of GPs reporting ignoring guidelines by offering testing to asymptomatic women (21, 22). Importantly, results from this study indicate that this practice may be misaligned with the benefits and harms offered by screening as patients only choose to accept screening when screening is stated to reduce ovarian cancer deaths. However, current screening modalities provide little-to-no benefit in terms of survival or stage of diagnosis, despite women’s perceptions that ovarian cancer screening leads to reduced mortality (23).

Similarly, a large proportion of the participants indicated they were unsure of their risk of ovarian cancer and did not feel confident in their ability to identify symptoms of ovarian cancer. Increasing education and awareness around ovarian cancer, encouraging help-seeking behaviour once symptoms arise and interventions to reduce mitigating lifestyle factors could provide a complementary (or alternative) strategy to improving ovarian cancer outcomes. These are particularly important given a universal screening programme may never be achievable.

### 4.2 Comparisons with other studies

The importance of ovarian cancer deaths follows trends seen in the broader screening DCE literature (16). The ability to make comparisons with findings from previous DCE studies is limited by the cancer site and the framing of the WTA calculations. In this study the MRS was expressed in terms of WTA increased deaths in exchange for improvements in risks, which is similar to the approach adapted by many of the studies in this area. Interestingly participants’ views of false positives are broadly within the higher and lower boundaries of other studies. For example, whilst this study finds that participants were willing to accept an additional 205 false positive results in exchange for one life saved, Sicsic et al. (24) estimated that women were willing to accept a lower rate of 47.8 (95% CI: 24.9-70.8) false positives per 1 breast cancer death avoided. Conversely, Schwartz et al. (25) estimated women were much more tolerant, with 63% believing that false positives of 500 or more per life saved was reasonable, and 37% willing to endure false positive rates of 10,000 in a cross-sectional survey directly eliciting the acceptability of false positive results in breast cancer.

On the other hand, overdiagnosed cancers appeared to be less tolerated within this study in comparison to Sicsic et al. (24), where on average 14.1 (95% CI:12.9-15.2) additional overdiagnosed cases were accepted in exchange for 1 breast cancer life saved. The overall trend towards decreased importance of overdiagnosis in screening relative to other attributes follows findings from other studies, including DCEs (26, 27). Most studies find individuals are sceptical or even hostile towards the concept of overdiagnosis, viewing the early detection of any cancer as a positive event (28, 29). Instead, studies typically find the concept of overtreatment to be of greater concern to participants (28, 30). There is further evidence of a compounding “cancer effect” whereby participants are more willing to endure risks of overdiagnosis when facing a possible diagnosis of cancer compared to other potentially serious and life limiting non-cancer conditions such as aortic aneurysms (31, 32).

Several studies support the findings that decisions to undergo screening may be driven by factors external to test efficacy or beliefs about the curability of the disease. Specifically, this study is in line with the findings from Bennet et al. (33) in which low perceived cancer risk, low cancer anxiety and increased confidence in the ability to spot symptoms all increase the likelihood of forgoing screening. Similarly, examination of test acceptability of participation enrolled on an ovarian cancer screening trial found high rates of self-perceived risk (34). More recently, de Bekker-Grob et al. (35) demonstrated similar findings within a DCE study, finding the 8-76% of non-participation behaviour in colorectal cancer screening was attributable to participant characteristics, particularly the individual’s attitude towards screening and previous screening behaviour, as opposed to the characteristics of specific tests.

## 5 Limitations

The attribute levels used in the study were fixed for all participants, and it was not possible to stratify attribute levels according to age or underlying risk of cancer of participants, due to lack of data on test performance in specific populations. Recent research suggests that the efficacy of tests may vary by age or underlying risk of cancer. For example, studies of cervical and breast screening have demonstrated the effectiveness of screening at reducing mortality varies with age (36–38). Testing of symptomatic women for possible ovarian cancer using CA125 tests has also been shown to be more effective for older patients perhaps due to the higher prevalence of cancer or the type of tumour (39). Similarly, screening of high-risk individuals is likely to be more effective due to higher incidence rates. Observed variations in preferences and willingness to trade across participants suggests any ‘one-size-fits all’ screening programme would fail to fulfil the priorities of the population overall. Examination of opt-out behaviour indicated that perceived risk of cancer plays an important role in screening decisions. Risk-stratified screening programmes or limiting screening to high-risk individuals only based on genetic and/or lifestyle factors may be potential solutions for implementation of screening programmes, in terms of acceptability, clinical efficacy and costs. Additionally, the current ineffectiveness of ovarian screening even within high-risk populations means screening is not routinely offered by the NHS. Risk-stratified screening is an ongoing and emerging area of research (40). Future studies aiming to understand the acceptability of, and preferences for such strategies for ovarian cancer are important.

Though trials of ovarian cancer screening have shown no reduction in mortality, trials have demonstrated a reduction in advanced stage disease, specifically in high-grade serious ovarian cancer (41–43). Diagnosis with less bulky advanced ovarian cancer still has its advantages for women, in that it would involve less radical surgery and perhaps with newer drugs, improved 5-year survival rates. Considerations of these issues was beyond the current study, but future studies could aim to elicit women’s views on this, to assess the important of reduction of advanced state over and above any change in long term mortality.

Although our study underwent a rigorous developed process, including public and patient involvement and think aloud interviews to check comprehension of the questions, it could be argued that women may still have lacked in-depth clinical and research knowledge to understand test performance characteristics. To assist women in understanding and interpreting the information presented, additional details were given about each test performance characteristic at the start of the questionnaire. In particular, information was given on the requirement for unnecessary surgery when receiving a false positive result in ovarian cancer screening, the potential for false reassurance following a false negative result and the potential for unnecessary treatment in overdiagnosis (see **Supplementary Material 1**). However, the study did not explicitly mention that unnecessary surgery may result in a small risk of serious complications. It would be interesting for this study to be replicated amongst health care providers that are likely to be well informed about test performance characteristics, to explore whether their preferences differ systematically from women.

This study focused on the importance of test performance characteristics based on the findings of a prioritisation best-worst scaling study. A review of clinical trials evidence formed the basis of attribute levels (17, 44) and level ranges were extended beyond currently observed efficacy levels to allow benefits of potential future screening tests to be evaluated (4). However, studies have demonstrated that public understanding of the test characteristics of screening programs is low, but screening is generally viewed favourably regardless (11, 12). These findings suggest that screening behaviours may be driven by service delivery factors that impact the convenience and overall experience of screening. Existing DCEs relating to cancer screening provide an extensive evidence base to draw from when considering the influence of service delivery attributes on cancer screening: however, given the low prevalence of ovarian cancer, willingness to endure inconveniences and disruptions associated with screening may be less tolerated, meaning an additional study may be of value, particularly once a viable screening modality emerges. Evidence from trials demonstrates the impact of test experience such as pain, embarrassment or inconvenience had very minimal (1-2%) impact on willingness on the acceptability and adherence of future screening (34). However, it remains unclear if this finding is transferable to the general population.

Finally, in our study participants ranged from 40 to 80 years old with an average age of 53 years. In terms of ethnicity, the sample included 20% non-white which is broadly similar to the Office of National Statistics 2021 survey of 18% for the UK population (45). However, a limitation of the study was that it included a relatively high proportion educated women, which potentially affects the generalizability of the results.

## 6 Conclusion

Currently ovarian cancer screening is not recommended as available screening methods do not offer any benefits in terms of mortality reduction. The results of this study provide a useful resource for assessing the acceptability of future screening modalities and for setting the false positives and false negative cut-off for novel screening tests that are in the pipeline. Overall, our results suggest the ability to reduce ovarian cancer deaths is the most important test performance characteristic; however, there was significant heterogeneity across participants.

## Data Availability

The raw data supporting the conclusions of this article will be made available by the authors, without undue reservation.
