# Supplementary material for: Exploring public preferences and demand for ovarian cancer screening: a discrete choice experiment
Source: Front Oncol. 2025 Apr 24;15:1467457. doi: 10.3389/fonc.2025.1467457 (PMC12058507; doi:10.3389/fonc.2025.1467457)
Supplement: Supplementary file 1 [file DataSheet1.pdf]

Supplementary Material

Exploring public preferences and demand for ovarian cancer screening: a discrete choice experiment

Rebekah Hall, Anne E Spencer*, Abigail Lloyd, Willie Hamilton, Antonieta Medina-Lara

*** Correspondence:** Corresponding Author: a.e.spencer@exeter.ac.uk

Supplementary material 1 Final version of the screening DCE survey instrument

Women's preferences towards ovarian cancer screening

INFORMATION SHEET FOR PARTICIPANTS

VERSION NUMBER [4]: DATE [02/12/21]

Thank you for showing an interest in this survey. Please take time to read the following information carefully before deciding whether or not to take part.

What is the aim of the project?
Ovarian cancer is the 6th most common cancer in women in the UK. A screening test for ovarian cancer (similar to cervical screening or 'smear test') could help to identify the disease earlier and improve survival outcomes. Research to develop a suitable screening test is ongoing but it important to make sure any potential test is acceptable to patients and the public. In this study we would like to understand the attitudes and preferences of womena and people with ovaries towards a hypothetical test for ovarian cancer. In particular, we want to learn what characteristics of diagnostic testing are most important.

Why have I been invited to take part?
We are approaching you because we are seeking responses from women and people with ovaries over the age of 40. You do not need to have any prior knowledge of ovarian cancer and you do not have to have been previously tested for ovarian cancer to take part in the study. You must be able to complete the survey in English to take part. **Please do not take part in this survey if you have ever undergone a procedure to remove both of your ovaries.**

What will I be asked to do?
Should you agree to take part, you will be asked to complete a survey lasting around 15 minutes. During the survey you will be shown descriptions of two different medical tests and asked which test you would prefer to have. In total, we will ask you about 6 pairs of tests. At the end of the survey we will ask you some additional questions about yourself. This will help us to better understand how attitudes might vary from person to person.

What are the possible disadvantages and risks of taking part?
Participating in the research is not anticipated to cause you any disadvantages or discomfort. Some questions may be considered sensitive, however, you do not have to provide responses to any questions you feel uncomfortable answering. During the survey you will be asked to imagine you might have cancer, for some this may cause anxiety.

Will I be paid?

You will receive a payment of £2.00 for completing the survey. Payments will be paid directly into your Prolific account within 10 working days.

Please note: In order to receive the payment you must complete the full survey and click the completion link at the end of the survey. To ensure responses are high quality, an “attention check” question has been included within the survey. You must correctly complete this question to receive payment.

Can I change my mind and withdraw from the project?
If you decide you no longer wish to take part during the survey, simply exit the webpage to withdraw. Your incomplete responses will be permanently deleted. If you decide to withdraw after submitting your responses, please contact us via your Prolific account or directly by email. You can withdraw from the study for up to 14 days after completion and do no not have to give a reason. After 14 days it may no longer be possible to withdraw your submission because anonymization will mean we cannot link responses to individual participants.

Is the survey confidential?
All your answers to the survey are completely confidential and anonymous. You will not be asked your name or any other identifying information. Your responses will be securely stored on an encrypted password protected computer and managed according to a law called the Data Protection Act (2018). Your anonymised data will be stored for a period of five years.

In line with the Cancer Research UK data sharing guidelines, your data may be shared with other researchers in the future at our discretion. Any shared data will be fully anonymised. For more information: https://www.cancerresearchuk.org/funding-for-researchers/applying-for-funding/policies-that-affect-your-grant/submission-of-a-data-sharing-and-preservation-strategy/data-sharing-guidelines

The results of the study may be published in academic journals or conferences but any included data will not be individually identifiable.

The University of Exeter processes personal data for the purposes of carrying out research in the public interest. The University will endeavour to be transparent about its processing of your personal data and this information sheet should provide a clear explanation of this. If you do have any queries about the University’s processing of your personal data that cannot be resolved by the research team, further information may be obtained from the University’s Data Protection Officer by emailing dataprotection@exeter.ac.uk or at www.exeter.ac.uk/dataprotection

What if I have any questions?
If you have any questions about our project, either now or in the future, please feel free to contact Rebekah Hall by emailing rh591@exeter.ac.uk

Complaints
If you have any complaints about the way in which this study has been carried out please contact the Chair of the College of Medicine and Health Research Ethics Committee:-

Mark Tarrant, PhD
Chair of the CMH Research Ethics Committee

Email: cmhethics@exeter.ac.uk

This project has been reviewed and approved by the University of Exeter College of Medicine and Health Research Ethics Committee (REF NUMBER: 20/09/261)

Consent Form

- I understand that my participation is voluntary and that I am free to withdraw for up to 14 days without giving any reason and without my legal rights being affected.

- I understand that my data from the study will be fully anonymised and will be looked at by members of the research team and may potentially be shared with other researchers in future if appropriate.

- I understand that relevant sections of the data collected during the study may be looked at by individuals from the University of Exeter, Cancer Research UK or regulatory authorities for audit purposes

- I understand that the results of the study may be published in academic journals but my anonymity will be preserved

- I understand that my anonymised data will be securely stored on an encrypted password protected computed for a period of five years.

- I understand that in order to receive payment for this survey I must complete the full survey and click the link at the end of the survey. I must also correctly complete an attention check question randomly placed within the survey.

I confirm that I have read the information above and agree to take part in the study:

☐ Yes

☐ No

Have you ever had a medical procedure that involved the removal of both of your ovaries?

○ Yes ○ No

Please enter your ProlificID

Next

Thank you for agreeing to take this survey

The survey will present some information about ovarian cancer and describe some tests. We will then ask you some questions about your health and about cancer testing. Later we will ask you to consider different tests for ovarian cancer.

Let's start with some information about ovarian cancer…

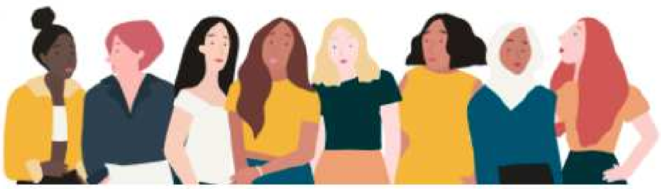

Next

Introduction to ovarian cancer screening

Ovarian cancer occurs when the cells in and around the ovaries and fallopian tubes become abnormal, grow out of control and form a lump called a "tumour".

Ovarian cancer is the 6th most common cancer for women in the UK. Over 7,000 women are diagnosed annually. Most of these cases occur in women over the age of 40.

Screening tests can help to identify certain types of cancer earlier, **before any symptoms arise**. Earlier diagnosis means more treatment options are available and can help to improve the chance of being cured or living longer.

In the UK screening tests are currently available for breast (mammogram), cervical ("smear test") and colorectal cancer.

There is currently no recommended screening test for ovarian cancer, however, research is ongoing and it is hoped a suitable test will be developed and approved in the future.

To be approved any screening test must be proven to save lives, however, all medical tests are also involve some risks (such as incorrect results or side effects). This means it is important to make sure any potential test meets the expectations of people who may be invited to have them and the balance of benefits and potential harms is acceptable to patients and the public.

In this survey we would like to find out the most important aspects of testing are most important to people with ovaries and how people balance the potential benefits (e.g. improved chance of survival) against potential harms.

Next

Ovarian cancer risk factors

Deciding whether to undergo screening is a personal decision and everyone is different.

Some people may find it helpful to have a better understanding of their risk of developing ovarian cancer before making a decision.

Without screening approximately 65 in 10,000 people will develop ovarian cancer over a 10-year period.

There is no way to know for sure who will develop ovarian cancer, however, there are some factors that increase or decrease the personal risk.

Factors that increase the risk of ovarian cancer:

- Family history of ovarian cancer
- Getting older
- Previous cancer diagnosis (especially if you were diagnosed before the age of 40)
- Using hormone replacement therapy (HRT)
- Smoking
- Obesity
- Certain medical conditions (e.g. diabetes, endometriosis)

Factors that may decrease the risk of ovarian cancer:

- Taking the combined contraceptive pill at some point in your life
- Having children and/or breastfeeding
- Having a hysterectomy or sterilisation ("tubes tied")

If you would like more information about risks of ovarian cancer please visit the Cancer Research website: https://www.cancerresearchuk.org/about-cancer/ovarian-cancer/risks-causes

Next

Part 1: Ovarian cancer knowledge

We would like to learn more about your knowledge and experience of ovarian cancer.

Have you ever undergone testing for possible ovarian cancer?

○ Yes

○ No

○ I don't know

○ Prefer not to say

Which of the following do you recognise as a symptom of ovarian cancer?

Check all that apply

- [ ] Feeling constantly bloated
- [ ] A swollen tummy
- [ ] Discomfort in your tummy
- [ ] Persistent indigestion or feeling sick
- [ ] Discomfort in your pelvic area
- [ ] A change in bowel habits
- [ ] Back pain
- [ ] Pain during sex
- [ ] Feeling full quickly or loss of appetite
- [ ] Feeling tired all the time
- [ ] Unintentional weight loss
- [ ] Needing to pee more often or more urgently than usual
- [ ] None

How confident are you that you would notice a symptom of ovarian cancer?

Not confident at all — 1 ——— 3 ——— 5 — Extremely confident

When was the last time you visited your GP?

This is an attention check question. Pease enter 'yes' to show that you are paying attention

Next

Part 2: Preferences towards ovarian cancer screening

During this section of the survey you will be asked to choose between screening tests which differ in terms of 4 characteristics:

1. Ovarian cancer deaths

2. False-positive results

3. False-negative results

4. Overdiagnosed cancers

These 4 characteristics are described in more detail on the next pages.

The rate at which these benefits and harms occur is described based on 10,000 people undergoing yearly screening over a period of 10-years. This will hopefully make it easier for you to compare the benefits and harms against each other.

In total, approximately 9 million people in England and Wales would be eligible for this hypothetical screening test (people with ovaries, aged 50-75 years old).

Next

1. Ovarian cancer deaths

This is the number of people who will die from ovarian cancer.

In this study, having no screening will lead to 40 deaths per 10,000 women over 50 years old.

For any screening test to be approved there must be strong evidence that the test reduces the number of deaths from ovarian cancer compared to no screening.

The screening tests you will be shown could reduce ovarian cancer deaths to:

- 30 deaths per 10,000 women over 50 years old
- 20 deaths per 10,000 women over 50 years old
- 10 deaths per 10,000 women over 50 years old

Next

2. False-positive results

These are people who do not have cancer but receive a positive (or abnormal) result.

People who receive an incorrect possible result will undergo unnecessary, often invasive testing.

A small proportion of these people (about 3%) will undergo unnecessary surgery because of the incorrect result.

Choosing not to be screened means there is no risk of false-positive results.

Over a 10-year period, the screening tests you will be shown in this study may result in:

- 1000 false-positive results per 10,000 women screened, leading to 30 unnecessary surgeries
- 2000 false-positive results per 10,000 women screened, leading to 60 unnecessary surgeries
- 3000 false-positive results per 10,000 women screened, leading to 90 unnecessary surgeries
- 4000 false-positive results per 10,000 women screened, leading to 120 unnecessary surgeries

Next

3. False-negative results

These are people who have cancer but receive a negative (or normal) result.

An incorrect negative result leads to false reassurance that they are disease-free and will mean diagnosis and treatment will be delayed.

Choosing not to be screened means there is no risk of false-negative results.

Over a 10-year period, the screening tests you will be shown in this study may result in:

- 3 false-negative results per 10,000 women screened
- 7 false-negative results per 10,000 women screened
- 10 false-negative results per 10,000 women screened
- 13 false-negative results per 10,000 women screened
- 16 false-negative results per 10,000 women screened
- 20 false-negative results per 10,000 women screened

Next

4. Overdiagnosed cancers

These are people who have cancer and are correctly diagnosed using the test. However, the cancer would never lead to death and may even never cause any symptoms.

These people will undergo unnecessary treatment.

Treatments for cancer (e.g. chemotherapy, radiation, surgery) have serious side-effects and often have long-term physical, mental and sometimes financial consequences (due to lost work).

Doctors are unable to tell which patients have a life-threatening disease and who has been over-diagnosed so everyone is offered treatment.

Choosing not to be screened means there is no risk of being over-diagnosed with ovarian cancer.

Over a 10-year period, the screening tests you will be shown in this study may result in:

- 0 cases of over-diagnosed cancer per 10,000 women screened
- 3 cases of over-diagnosed cancer per 10,000 women screened
- 7 cases of over-diagnosed cancer per 10,000 women screened
- 11 cases of over-diagnosed cancer per 10,000 women screened
- 16 cases of over-diagnosed cancer per 10,000 women screened

Next

Shortly you will be asked to complete 12 questions comparing test options.

Each question will have 3 options to choose from: Test A, Test B or no screening test.

Each option is described by the 4 characteristics previously described.

The questions will look like this:

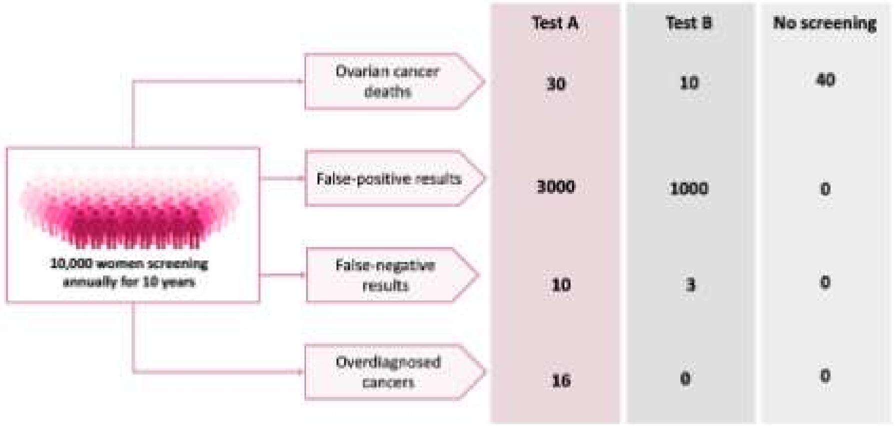

For each question, please look at each option and decide which option you would choose if you were asked to make this decision in real life.

These questions are designed to reflect real medical decisions which can be difficult to consider.

There are no right or wrong answers. We are interested in your opinion.

Next

Warm up question

To help you become familiar with the format of the questions, please answer the warm up question below

If you were given the choice between the options below, which would you choose?

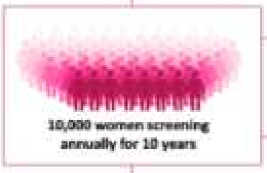

	Test A	Test B	No screening
Ovarian cancer deaths	20	10	40
False-positive results	2000	1000	0
False-negative results	10	3	0
Overdiagnosed cancers	7	0	0

- Test A
- Test B
- I would choose not to be screened

Use your mouse to hover over the characteristics below if you would like a reminder of the characteristic definitions

Ovarian cancer deaths

False-positive results

False-negative results

Overdiagnosed cancers

Next

Great! You will not be asked twelve more questions comparing different test options using the same question style.

Remember there are no right or wrong answers.

Next

12 DCE QUESTIONS HERE

In this next section you will be asked some follow-up questions about your decision process in the last section. Again, there are no right or wrong answers.

How easy or difficult did you find making your choices?

- ○ Very easy
- ○ Easy
- ○ Neither easy or difficult
- ○ Difficult
- ○ Very difficult

Which characteristics did you consider when making your choices?.

- ☐ Ovarian cancer deaths
- ☐ False-positive results
- ☐ False-negative results
- ☐ Overdiagnosed cancers

What characteristics did you ignore when making your choices?

- ☐ Ovarian cancer deaths
- ☐ False-positive results
- ☐ False-negative results
- ☐ Overdiagnosed cancers
- ☐ I did not ignore any of the characteristics

Why did you ignore certain characteristics?

- [x] There were too many characteristics to look at
- [ ] The other characteristics were unclear
- [ ] The other characteristics were not important to me
- [ ] Other:

You indicated that you would choose not to be screening in any of the scenarios. Please could you briefly explain this decision?

Please rank the four characteristics from most to least important:

Your choices	Your ranking
Overdiagnosed cancers	
Ovarian cancer deaths	
False-negative results	
False-positive results	

Double-click or drag-and-drop items in the left list to move them to the right - your highest ranking item should be on the top right, moving through to your lowest ranking item.

Next

Part 3: Background questions

In the last part of the survey we want to learn a bit more about you and your background. Your answers will be used to understand how preferences towards ovarian cancer testing might vary between different people.

What is your age?

[ ]

What is your ethnicity?

- ○ Prefer not to say
- ○ White/Caucasian
- ○ Mixed- white and black African
- ○ Mixed- white and black Caribbean
- ○ Mixed- White and Asian
- ○ Asian- Indian
- ○ Asian- Bangladeshi
- ○ Asian- Chinese
- ○ Black-African
- ○ Black- Caribbean
- ○ Arab
- ○ Other: [ ]

Which of the following best describes your current relationship status?

- ○ Single
- ○ In a relationship
- ○ Married/In a registered civil partnership
- ○ Separated/divorced
- ○ Widowed
- ○ Prefer not to say

How many children do you have?

[ ]

Which of the following categories best describes your employment status?

[Please choose... ▾]

What is the total annual income of your household (before tax)?

- Prefer not to say
- £0-£9,999
- £10,000-£19,999
- £20,000-£29,999
- £30,000-£39,999
- £40,000-£49,999
- £50,000-£59,999
- £60,000-£69,999
- £70,000 or more

What is the highest level of education you have completed?

Please choose...

Next

How is your health in general?

- Very good
- Good
- Fair
- Bad
- Very bad
- Prefer not to say

Compared to the average woman of your age, how would you describe your risk of developing ovarian cancer?

- Very high risk
- High risk
- Average risk
- Low risk
- Very low risk
- Prefer not to say
- Don't know

To what extent do you worry about your risk of ovarian cancer?

- Not at all
- A little
- A moderate amount
- A lot
- A great deal
- Prefer not to say

On average, how often do you visit your GP every year? Please enter 'yes' to show that you are paying attention

Have you ever been diagnosed with any type of cancer?

- Yes
- No
- Prefer not to say

To your knowledge, have any of your family or friends been diagnosed with ovarian cancer?

- Yes
- No
- Prefer not to say

Was this person your:

Check all that apply

- [ ] Blood relative
- [ ] Non-blood relative
- [ ] Friend
- [ ] Acquaintance/work colleague
- [ ] Prefer not to say
- [ ] Other: ________

Do you regularly attend cervical screening (smear test) appointments?

- ○ I attend every time I receive an invitation
- ○ I attend cervical screening sometimes
- ○ I used to attend regularly but have stopped
- ○ I have never attended

Regularly means whenever you receive an invitation letter in the post. This is every 3 years if you are under 45 years old or every 5 years if you are under 64 years old.

In general, how willing are you to take risks?

Completely unwilling — 5 — Completely willing

1 … 10

Next

Part 4: Background questions

In this final section you will be asked some questions to understand how comfortable you are with probabilities.

These are not attention check questions. You will still receive payment even if you answer incorrectly.

The PSA (prostate specific antigen) is a blood test that looks for prostate cancer. The test has false alarms so about 30% of men who have an abnormal test turn out not to have prostate cancer. John had an abnormal test. What is the chance that John has prostate cancer?

- 0%
- 30%
- 70%
- 100%

James starts a new blood pressure medicine. The chance of a serious side effect is 0.5%. If 1000 people take this medicine, about how many would be expected to have a serious side effect?

- 1 person
- 5 people
- 50 people
- 500 people

A medical study will randomly assign people so that people are equally likely to get medicine A or medicine B. If there are 300 people in the study, about how many are expected to get medicine A?

- 100 people
- 150 people
- 200 people
- 250 people
- I am not sure

Natasha started a new medicine and was given a handout showing the chance that side effects will occur. Which side effect is Natasha least likely to get?

- a. Dizziness: 1 in 5 people
- b. Nausea: 1 in 10 people
- c. Stomach pain: 1 in 100 people
- d. Allergic reaction: 1 in 200 people
- I am not sure

Amanda is told she has a 1 in 296 chance of dying from cancer and a 1 in 407 chance of dying from a stroke. 6. Which is bigger, Amanda chance of dying from a stroke or cancer?

- Stroke
- Cancer
- Chances are the same
- I am not sure

Next

If you have any additional comments about any of the questions or comments about the survey you have just completed, please leave them below:

Submit

Thank you for completing this survey.

Important: Please click here to return to prolific and confirm your submission

---

Your responses to this survey will add to a body of research which we hope to understand public priorities around a potential future test for ovarian cancer. Currently screening for ovarian cancer is not reccomended since evidence from large trials suggests there is no benefit to screening for ovarian cancer. However, research is ongoing and promising new developments have been made in recent years. This information from this survey will be useful in tailoring ongoing research and policy decisions in the development of screening tests.

We know that cancer is a sensitive subject and being asked to imagine the scenarios we have shown during the survey may have cause some people to feel anxious or concerned. Please speak to your GP if you are concerned about your risk of ovarian cancer.

As with most cancers, early recognition of symptoms will help increase the chances of sucessful treatment. Being of aware of the symptoms will help you to spot them more easily.

Common symptoms of ovarian cancer include:

- feeling constantly bloated
- a swollen tummy
- discomfort in your tummy or pelvic area
- feeling full quickly when eating
- needing to pee more often than usual

Please contact your GP if you have any of these symptoms and do not go away. More information on ovarian cancer can be found on the NHS website: https://www.nhs.uk/be-clear-on-cancer/symptoms/ovarian-cancer

More information about ovarian cancer and the tests available can be found at :

Cancer Research UK: https://www.cancerresearchuk.org/about-cancer/ovarian-cancer

Target ovarian cancer: https://www.targetovariancancer.org.uk

The Eve Appeal: https://eveappeal.org.uk/gynaecological-cancers/ovarian-cancer/

If you are currently living with ovarian cancer and have been affected by any of the issues in this study, please speak to your clinical nurse specialist.

If you have any questions or concerns about the survey please contact Rebekah Hall at rh591@exeter.ac.uk or Prof Anne Spencer at A.E.Spencer@exeter.ac.uk

Postal address: University of Exeter Medical School, Room 1.15, South Cloisters, St Luke's Campus, Magdalen Road, City, Exeter, EX1 2LU

If you would like to be kept informed of the results from this study, please contact Rebekah Hall via your prolific account.

Supplementary material 2: Risk communication

Figure 1: Adapted probability tree

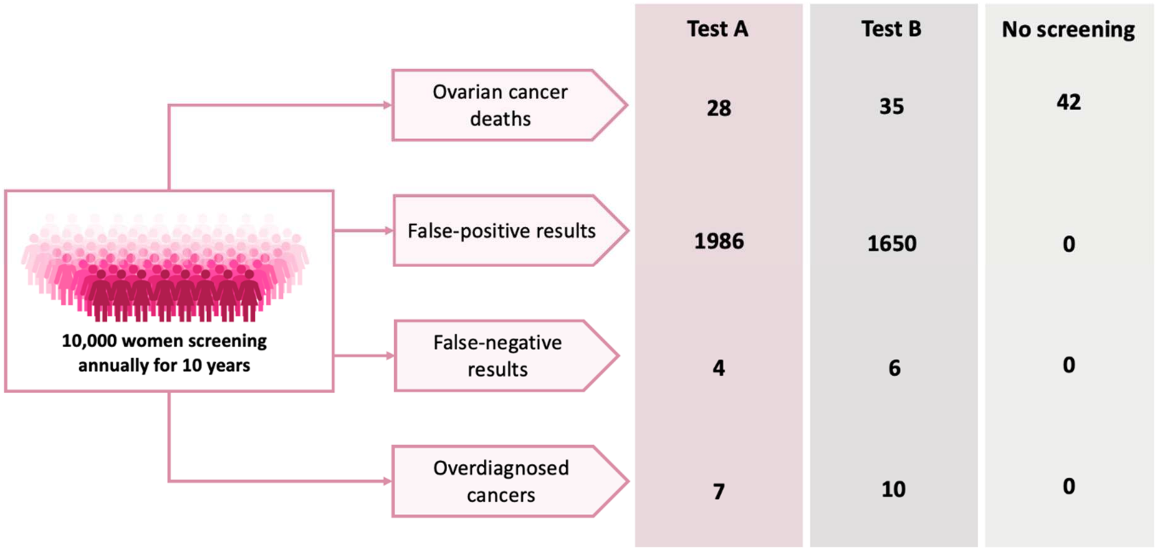

Supplementary material 3: Health-related characteristics of respondents completing the ovarian cancer screening DCE survey

Characteristic	
**Perceived risk of ovarian cancer, n (%)**	
Very low	12 (5%)
Low	45 (18%)
Average	149 (60%)
High	22 (9%)
Very high	1 (0.4%)
Don't know	21 (8%)
**Ovarian cancer-related worry, n (%)**	
A great deal	6 (2%)
A lot	13 (5%)
A moderate amount	46 (18%)
A little	108 (44%)
Not at all	76 (30%)
**Confidence to recognise OC symptoms, n (%)**	
1-Not at all	74 (30%)
2	118 (47%)
3	36 (14%)
4	20 (8%)
5-Extremely confident	2 (1%)
**Symptom recognition, n (%)**	
Feeling constantly bloated	149 (60%)
Swollen tummy	143 (57%)
Discomfort in your tummy	136 (54%)
Persistent indigestion or feeling sick	55 (22%)
Discomfort in your pelvic area	169 (68%)
A change in bowel habits	77 (31%)
Back pain	105 (42%)
Pain during sex	103 (41%)
Feeling full quick or loss of appetite	76 (30%)
Feeling tired all the time	118 (47%)
Unintentional weight loss	151 (60%)
Needing to urinate more often or more urgently that usual	85 (34%)
None	29 (12%)
**Personal history of cancer, n (%)**	22 (9%)
**Knew someone who was diagnosed with ovarian cancer, n (%)**	41 (16%)
**Previously tested for ovarian cancer, n (%)**	26 (10%)
**Cervical cancer screening attendance, n (%)**	
Attends every time	157 (63%)
Attends sometimes	35 (14%)
Used to attend but stopped	47 (19%)
Never attended	11 (4%)
**Breast cancer screening attendance, n (%)**	
Attends every time	52 (21%)
Attends sometimes	3 (1%)
Used to attend but stopped	13 (5%)
Never attended	13 (5%)
Not eligible	169 (68%)
**Self-reported overall health, n (%)**	
Very good	34 (14%)
Good	124 (50%)
Fair	77 (31%)
Poor	14 (6%)
Very poor	1 (0.4%)

Supplementary material 4: Mixed logit model excluding respondents who failed the rationality check (n=12)

	Coeff.	95% confidence interval	SD
Ovarian cancer deaths	-0.14***	-0.12 – [-0.16]	0.10***
False negatives	-0.06***	-0.04 – [-0.07]	0.05***
False positives	-0.00***	-0.00 – [-0.00]	0.00***
Overdiagnosed cancers	-0.06***	-0.05 – [-0.07]	0.04***
Neither test	-2.33***	-1.57 – [-3.02]	5.31***
**Model fit statistics**			
LL	-1803.12		
Observations	8,568		
N	238		
Key: ***significant at 99% confidence level; **significant at 95% confidence level; *significant at 90% confidence level			
